# Similarities and Differences Between HFmrEF and HFpEF

**DOI:** 10.3389/fcvm.2021.678614

**Published:** 2021-09-20

**Authors:** Peixin Li, Hengli Zhao, Jianyu Zhang, Yunshan Ning, Yan Tu, Dingli Xu, Qingchun Zeng

**Affiliations:** ^1^State Key Laboratory of Organ Failure Research, Department of Cardiology, Nanfang Hospital, Southern Medical University, Guangzhou, China; ^2^Guangdong Provincial Key Laboratory of Shock and Microcirculation, Southern Medical University, Guangzhou, China; ^3^Bioland Laboratory (Guangzhou Regenerative Medicine and Health Guangdong Laboratory), Guangzhou, China; ^4^School of Laboratory Medicine and Biotechnology, Southern Medical University, Guangzhou, China; ^5^Department of Cardiology, Foshan First People's Hospital, Foshan, Guangdong, China

**Keywords:** heart failure, heart failure with mid-range ejection fraction, heart failure with preserved ejection fraction, heterogeneity, cardiac abnormality

## Abstract

The new guidelines classify heart failure (HF) into three subgroups based on the ejection fraction (EF): HF with reduced EF (HFrEF), HF with mid-range EF (HFmrEF), and HF with preserved EF (HFpEF). The new guidelines regarding the declaration of HFmrEF as a unique phenotype have achieved the goal of stimulating research on the basic characteristics, pathophysiology, and treatment of HF patients with a left ventricular EF of 40–49%. Patients with HFmrEF have more often been described as an intermediate population between HFrEF and HFpEF patients; however, with regard to etiology and clinical indicators, they are more similar to the HFrEF population. Concerning clinical prognosis, they are closer to HFpEF because both populations have a good prognosis and quality of life. Meanwhile, growing evidence indicates that HFmrEF and HFpEF show heterogeneity in presentation and pathophysiology, and the emergence of this heterogeneity often plays a crucial role in the prognosis and treatment of the disease. To date, the exact mechanisms and effective treatment strategies of HFmrEF and HFpEF are still poorly understood, but some of the current evidence, from observational studies and *post-hoc* analyses of randomized controlled trials, have shown that patients with HFmrEF may benefit more from HFrEF treatment strategies, such as beta-blockers, angiotensin-converting enzyme inhibitors, angiotensin receptor blockers, mineralocorticoid receptor antagonists, and sacubitril/valsartan. This review summarizes available data from current clinical practice and mechanistic studies in terms of epidemiology, etiology, clinical indicators, mechanisms, and treatments to discuss the potential association between HFmrEF and HFpEF patients.

## Introduction

In 2016, heart failure (HF), a clinical syndrome with typical signs and symptoms triggered by a structural and/or functional cardiac abnormality resulting in reduced cardiac output (CO) and/or elevated intracardiac pressures at rest or during stress, was categorized into three subgroups based on left ventricular ejection fraction (LVEF): HF with reduced EF (HFrEF; EF < 40%), HF with mid-range EF (HFmrEF; EF 40–49%), and preserved EF (EF ≥ 50%) (1). Compared with the previous guidelines, this new one identified HFmrEF as a unique phenotype that has been stimulating more research into the clinical characteristics, pathophysiology, and treatment of HFmrEF populations (2).

Data from the ESC Heart Failure Long-Term Registry showed that no significant difference was found in all-cause mortality between HFmrEF and HFrEF or HFpEF, while the mortality rate among HFrEF patients was markedly higher than that among HFpEF patients. Non-cardiovascular (CV) mortality was numerically higher in patients with HFmrEF and HFpEF than in those with HFrEF. The incidences of 1-year death and hospitalization for HF among HFmrEF and HFpEF patients were less than those among HFrEF patients (3, 4). Additionally, in a pooled analysis of cohorts from three Northwestern European countries, patients with HFmrEF and HFpEF had similar survival rates, both of which were better than those for HFrEF patients (4, 5). In the APOLLON trial, an observational and multicenter study completed in Turkey (6, 7), the HFmrEF patients were more likely to have ECG abnormalities and a history of hospitalization due to HF in the last year, while a lower frequency of palpitations was observed in HFpEF patients.

Therefore, in this review, we briefly describe the existing knowledge on the epidemiology, etiology, and clinical indicators of HFpEF and HFmrEF and discuss the underlying mechanisms of these two subgroups. Finally, we present the evidence on current potential treatment options for these two groups of patients.

## Epidemiology

In some registries and clinical trials, a significant proportion of patients with HF belong to the HFmrEF and HFpEF subgroups, with percentages ranging 8.1–24 and 11–43%, respectively (3–5, 8, 9)—for example, in the HF-PATHWAYS study in Spain, 19,762 patients with HF were identified out of 1,189,003 patients treated from 2017 to 2019, and the distribution of LVEF was as follows: 51.7, 8.1, and 40.2% were classified as having HFrEF, HFmrEF, and HFpEF, respectively (8). In the ESC HF Long-Term Registry, including all regions of European and Mediterranean countries, HFrEF, HFmrEF, and HFpEF accounted for 59.8, 24.2, and 16.0%, of the cases, respectively, among 9,134 patients (3). Similarly, in a trial of 169 participating hospitals in China from 2017 to 2018, 11,034 (35.2%) HFrEF patients, 6,825 (21.8%) HFmrEF patients, and 13,497 (43.0%) HFpEF patients were enrolled (5). In a Swedish registry, 4,942 patients were identified, 18%, 19%, and 63% of whom had HFpEF, HFmrEF, and HFrEF, respectively (9). In another pooled analysis from the Northwestern European cohorts, 10,312 patients with stable HF were enrolled, including 7,080 (68.7%) with HFrEF, 1,146 (11.1%) with HFpEF, and 2,086 (20.2%) with HFmrEF (4).

## Etiology

Although patients with HFrEF and HFpEF share many similar risk factors (10), some comorbidities differ between them. The HFpEF patients are more likely to be older (3, 5), men are more likely to have HFrEF, and women are predisposed to HFpEF (3, 5, 11). This can be attributed to women, compared with men, being more likely to suffer from the risk factors for HF, such as obesity, diabetes, mental/psychological stress, and socioeconomic deprivation (11). Additionally, men are more likely to have concentric hypertrophy, while women are more likely to suffer from eccentric hypertrophy (12). Patients with HFpEF have a higher comorbidity burden, including hypertension, atrial fibrillation/atrial flutter (AF), anemia, and chronic obstructive pulmonary disease (COPD) (3, 5) but are less likely to have ischemic etiology and left bundle branch block than HFrEF patients (3). Generally, patients with HFmrEF are more often described as an intermediate population between HFrEF and HFpEF patients. Nevertheless, the HFmrEF group resembled the HFrEF group with regard to age, sex, systolic blood pressure, and ischemic etiology but had less left ventricular (LV) and atrial dilation (3, 13); however, the most striking similarity between HFmrEF and HFrEF is that both are associated with a higher incidence of coronary artery disease and a greater risk of new ischemic heart disease than HFpEF (14). In addition, the HFmrEF and HFrEF populations had higher rates of previous myocardial infarction (MI) and AF than those with HFpEF (7, 13), whereas no significant differences were found in diabetes, chronic renal failure, or mean hemoglobin level between the HFmrEF and HFrEF groups (15). Additionally, regarding the history of hypertension, distribution of New York Heart Association class (NYHAC), and body mass index, the HFmrEF subgroup fell between the HFrEF and HFpEF groups (13) (Table 1).

**Table 1:** Baseline characteristics of patients with HFmrEF and HFpEF.

**Characteristics**	**Chioncel et al**. **(****3****)**		**Wang et al**. **(****5****)**		**Tsuji et al**. **(****14****)**		**Lund et al**. **(****13****)**		**Moliner et al**. **(****15****)**	
	**HFmrEF**	**HFpEF**	**HFmrEF**	**HFpEF**	**HFmrEF**	**HFpEF**	**HFmrEF**	**HFpEF**	**HFmrEF**	**HFpEF**
	***n*** **= 2,212**	***n*** **= 1,462**	***n*** **= 6,825**	***n*** **= 13,497**	***n*** **= 596**	***n*** **= 2,154**	***n*** **= 1,322**	***n*** **= 1,953**	***n*** **= 134**	***n*** **= 135**
Age (years)	64.2 ± 14.2	68.6 ± 13.7	67.7 ± 13.3	71.3 ± 12.5	69.0 ± 11.6	71.7 ± 10.9	65 ± 11	67 ± 11	67 ± 12	69.6 ± 14.4
Female gender (%)	31.5	47.9	35.2	50.2	28.2	39.2	29.9	45.5	32.1	60.7
BMI (kg/m^2^)	28.6 ± 5.4	28.4 ± 5.4	24.0 ± 4.0	24.2 ± 4.2	22.8 ± 5.3	23.2 ± 4.7	27.8 (25.0–31.2)	28.6 (25.4–32.6)	28.6 ± 5.4	28.6 ± 5.4
SBP (mmHg)	126.5 ± 21.1	130.98 ± 21.4	131.3 ± 22.4	134.9 ± 22.9	124.7 ± 19.3	127.9 ± 19.2	130 (120–145)	140 (124–150)	132.4 ± 20.6	135.4 ± 25.7
NYHA class III–IV, *n* (%)	18.4	20.3	36.2	41.6	11.7	10.6	42.3	38.9	28.4	34.8
IHD (%)	41.8	23.7	60.8	57.1	52.9	44.1	66.9	50.4	54.5	13.3
DCM (%)	27.6	11.6	10.6	2.9	20.3	6.4			6.7	1.5
Hypertension (%)	9.6	18.1	59.4	64.5	89.8	91.2	56.2	68.7	66.4	74.8
AF (%)	22.3	32.2	33.0	40.9	43.5	51.8	25.6	31.3	22.4	38.5
DM (%)	30.5	29.3	30.7	28.5	36.1	33.8	28.6	28.1	29.9	37.8
MI			39.4	22.4	41.1	26.9	57.6	37.0		
COPD (%)	11.6	14.0	7.4	9.6						
Prior stroke/TIA (%)	8.3	9.8	14.6	17.5	22.1	21.9	9.3	8.4		
Chronic kidney disease (%)	16.5	19.9	11.9	11.0						
LBBB (%)	15.4	8.7	5.0	2.3						
Anemia			28.5	31.7						

*AF, atrial fibrillation/flutter; BMI, body mass index; COPD, chronic pulmonary obstructive disease; DCM, dilated cardiomyopathy; DM, diabetes mellitus; IHD, ischemic heart disease; LBBB, left bundle branch block; MI, myocardial infarction; NYHA, New York Heart Association; SBP, systolic blood pressure; TIA, transient ischemic attack*.

## Clinical Indicators

Higher laboratory parameters, including blood urea nitrogen, creatinine, N-terminal pro-B-type natriuretic peptide (NT-proBNP), potassium, uric acid, and ferritin levels, were shown in populations of HFmrEF compared with HFpEF. Compared with the HFpEF patients, the HFmrEF patients had larger LV end-diastolic and end-systolic dimensions, a higher left atrial volume index and LV mass index, and a lower LVEF. Patients in the HFmrEF and HFpEF groups did not differ significantly in valvular disease (except for mitral regurgitation, from which the former group is more likely to suffer) or diastolic dysfunction parameters (6). A Swedish study assessed the association between NT-proBNP and CV vs. non-CV events in three subgroups of HF and found that the median NT-proBNP values in HFpEF and HFmrEF were similar but much lower than those in HFrEF. The occurrence of CV risk increased with lower EF values, while the occurrence of non-CV risk increased with higher EF values, and the CV–nonCV event ratio was positively correlated with the NT-proBNP concentration (16). In a panel of 37 biomarkers from different pathophysiological domains [e.g., myocardial stretch, oxidative stress (OS), inflammation, angiogenesis, and hematopoiesis], HFrEF was most related to cardiac stretch, HFpEF to cardiac inflammation, and HFmrEF to both stretch and inflammation (17). Another study in Spain consisting of a series of biomarkers, including NT-proBNP, neprilysin, galectin-3, soluble suppression of tumorigenesis-2 (sST2), high-sensitivity troponin T (hs-TnT), cystatin-C, soluble transferrin receptor (sTfR), and high-sensitivity C-reactive protein (hs-CRP), revealed that HFmrEF was quite similar to HFrEF because there were no differences in other measured biomarkers in HFmrEF and HFrEF, except that the level of NTproBNP was similar to that of HFpEF but was significantly decreased in HFrEF. When the HFmrEF patients were compared with the HFpEF patients, the HFmrEF patients had significantly lower levels of ST2 and cystatin C. The authors of the study also proposed that, except for galactose lectin-3 and neprilysin, all biomarkers of HFmrEF had a higher risk prediction ability than HFrEF or HFpEF, and only soluble neprilysin showed a superior prognostic value in patients with HFpEF than HFrEF and HFmrEF (15) (Table 2).

**Table 2:** Clinical indicator of patients with HFmrEF and HFpEF.

**Biomarkers**	**Ozlek et al. (6)**		**Moliner et al. (15)**		**Tromp et al. (17)**	
	**HFmrEF**	**HFpEF**	**HFmrEF**	**HFpEF**	**HFmrEF**	**HFpEF**
	***n*** **= 246**	***n*** **= 819**	***n*** **= 134**	***n*** **= 135**	***n*** **= 128**	***n*** **= 108**
BUN	H*	L*	NA	NA	L	H
Creatinine	H*	L*	NA	NA	NA	NA
NT-proBNP	H*	L*	H	L	H*	L*
Uric acid	H*	L*	NA	NA	NA	NA
Ferritin	H*	L*	NA	NA	NA	NA
Neprilysin	NA	NA	L	H	NA	NA
Galectin-3	NA	NA	L	H	L	H
sST2	NA	NA	L*	H*	L	H
hs-TnT	NA	NA	L	H	NA	NA
sTfR	NA	NA	L	H	NA	NA
hs-CRP	L	H	L	H	L*	H*
Cystatin C	NA	NA	L*	H*	NA	NA

BUN, blood urea nitrogen; hs-CRP, high-sensitivity C-reactive protein; hs-TnT, high-sensitivity troponin T; NT-proBNP, N-terminal pro-B-type natriuretic peptide; sTfR, soluble transferrin receptor; sST2, soluble suppression of tumorigenesis-2; H, higher—P > 0.05; L, lower—P > 0.05; H*, higher—P < 0.05; L*, lower—P < 0.05.

## Underlying Mechanisms of HFpEF and HFmrEF

Extensive data are lacking regarding the mechanism of HFmrEF. Therefore, we speculate on the underlying mechanism of HFmrEF, relying on current clinical registries and investigations that have reported the etiology and comorbidities of different EF values.

However, numerous HFpEF mechanistic studies have been performed recently, including those on coronary microvascular dysfunction centered on inflammation and endothelial damage and energy production disturbance and myocardial metabolic abnormalities centered on mitochondrial injury as well as diastolic dysfunction related to myocardial fibrosis and calcium homeostasis disorder.

Therefore, in the following section, we discuss the underlying mechanism of HFmrEF and HFpEF from inflammation, cardiomyocyte injury, endothelial dysfunction, cardiac fibrosis, Ca^2+^ homeostasis, and mitochondrial dysfunction.

### Inflammation

Since the new HFpEF paradigm of coronary microvascular inflammation was proposed in 2013, more preclinical and clinical data have emerged (18). The new paradigm in HFpEF results from a series of factors consisting of the following: (1) comorbidities, (2) reactive oxygen species, (3) limited NO bioavailability, (4) low protein kinase G (PKG) activity, and (5) diastolic dysfunction (19).

Extracardiac metabolic inflammation, such as overweight/obesity, diabetes, COPD, and hypertension, plays an important role in the pathogenesis of both HFpEF (19) and HFmrEF patients. In obese and type 1 and 2 diabetic patients, elevated levels of advanced glycation end products (AGEs) (20, 21) bind to their receptor, triggering the downstream NFκB signaling pathway and thereby promoting the secretion of adhesion molecules, chemokines, and proinflammatory cytokines (19); moreover, the concentrations of ST2 and cystatin-C were significantly higher in patients with HFpEF than in those with HFmrEF (15). However, tissue damage and necrosis (recognized as danger-associated molecular patterns, DAMPs) caused by ischemia, such as myocardial infarction, will lead to the release of cardiac antigens, which, in turn, induces local and systemic inflammatory responses (22) that are more common in HFmrEF. Simmonds et al. summarized this response as sterile inflammation induced by (a) exposure to host-derived non-microbial stimuli released through tissue injury and activation of the pathogen recognition receptor (PRR) pathway in a response called DAMPs, such as DNA, ATP, and hyaluronan, (b) activation of common pathways downstream of PRRs *via* released intracellular cytokines, and/or (c) activation of pathways unrelated to microbial recognition receptors, such as cluster of differentiation 36 (23–25).

These findings suggest that extracardiac metabolic inflammation may play a more important role in HFpEF, while sterile inflammation caused by ischemia appears more important in patients with HFmrEF.

### Endothelial Dysfunction

Endothelial cells constitute the majority of non-cardiomyocytes (>60%); their structural and/or functional abnormalities will strongly affect cardiac function, especially in HFpEF (26). Endothelial dysfunction is an early event in CV disease progression in HFpEF patients as opposed to the late-stage symptom that is often present in patients with HFrEF (19). Some complications, such as diabetes, can affect endothelial function to varying degrees, especially considering that the patients with HFpEF are more likely to suffer from metabolic complications, and the risk of endothelial dysfunction was more likely to be prevalent in patients with HFpEF than HFrEF (27). HFpEF myocardial biopsies confirmed this theory; compared with HFrEF patients, the bioavailability of NO decreased, and the uncoupling of eNOS increased (28). The imbalance of NO bioavailability and OS leads to the decreased endothelium-dependent vasodilatory function of coronary arteries (29–31), resulting in reduced myocardial perfusion and impaired coronary blood flow (29). The reduced NO results in decreased activities of cyclic guanosine monophosphate (cGMP) and PKG and increased activities of protein phosphatase 1 and 2a (28). Downregulating NO-cGMP-PKG signaling may be attributed to cardiomyocyte hypertrophy and stiffness in HFpEF.

However, the role of endothelial dysfunction in the pathophysiology of HFmrEF has not yet been reported, and further study is needed. Another study demonstrated that the levels of endothelial dysfunction biomarkers (endothelin-1 and E-selectin) were interrelated with EF (32).

### Cardiomyocyte Injury

In the pathophysiology of HFpEF, growing evidence shows that coronary microvascular dysfunction leads to cardiomyocyte injury, especially in women with cardiometabolic risk factors and LVH (18). In a research on multiple biomarkers in Spain, Moliner et al. showed that the TnT level, a typical biomarker reflecting cardiomyocyte injury, in HFmrEF patients was twofold higher than those in HFrEF and HFpEF patients (15). Combined with its ischemic etiology, it is plausible that cardiomyocyte injury, but not cardiomyocyte death, may play a greater role in patients with HFmrEF than HFpEF. Additionally, several studies have demonstrated elevations in troponin I (TnI) (33) and TnT (34) in patients with HFpEF. Obokata et al. (34) found that the TnT levels were elevated in HFpEF both at rest and during exercise, and the extent of the rise was directly related to the increase in left ventricular filling pressure due to the lower myocardial oxygen supply and the imbalance of myocardial oxygen supply–demand. These findings suggested that cardiomyocyte injury was involved in the development of both HFpEF and HFmrEF patients and may provide a novel therapeutic target for this syndrome.

### Diastolic Dysfunction Associated With Cardiac Fibrosis and Ca^2+^ Homeostasis

**Figure 1:**
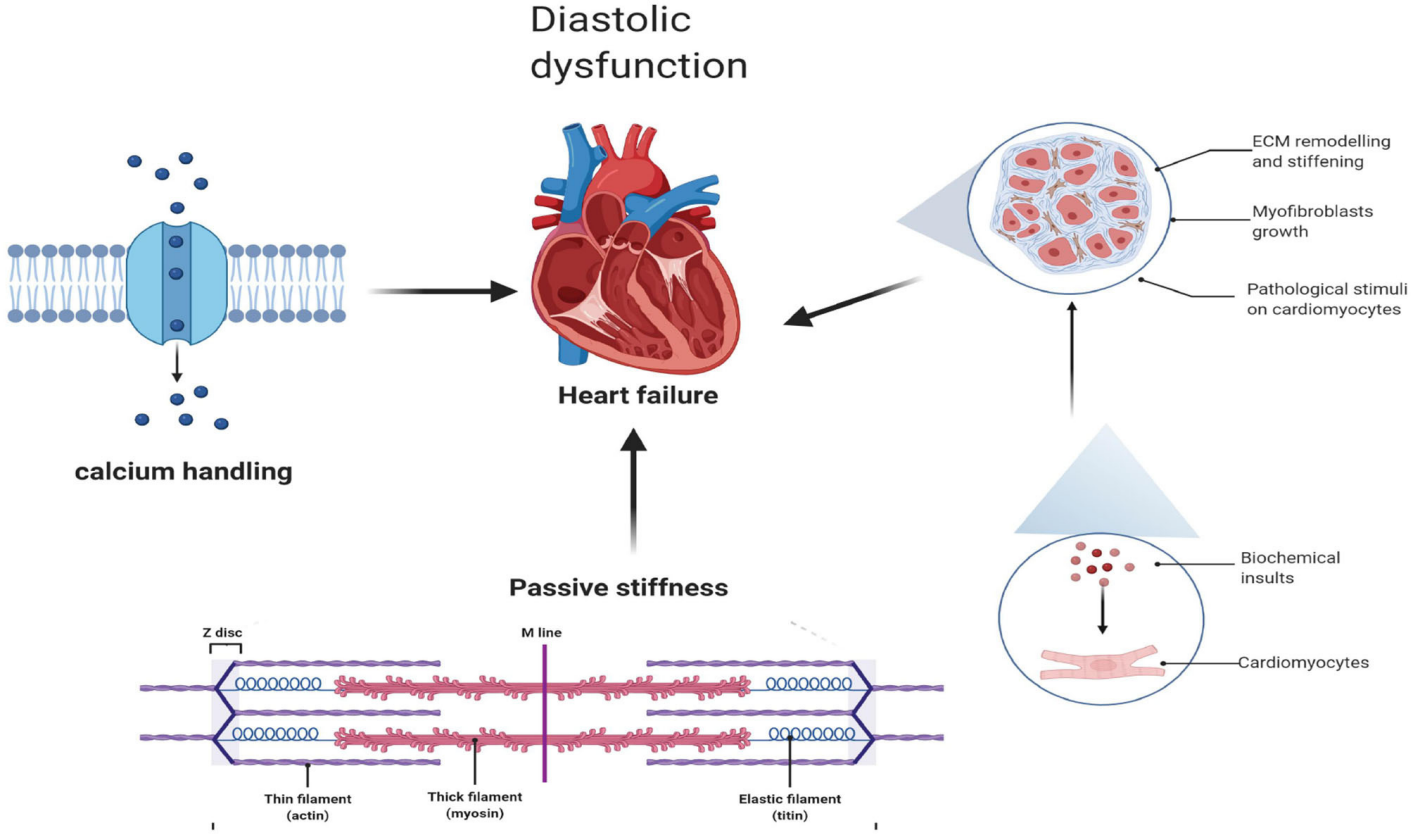
Diastolic dysfunction associated with cardiac fibrosis and Ca^2+^ homeostasis.

Diastolic dysfunction, as a hallmark of HFpEF, is triggered by abnormalities in excitation–contraction coupling (35) and ventricular stiffness (36), while cardiac fibrosis and abnormalities in active relaxation and passive stiffness (F-passive) (37) mainly affect ventricular stiffness and, thus, decrease ventricular filling, elevating ventricular pressures during diastole (38) (Figure 1).

#### Cardiac Fibrosis

Myocardial fibrosis, as an important pathophysiological mechanism, plays a key role in the emergence and progression of the disease, regardless of EF (39). In patients with HFmrEF and HFpEF, myocardial fibrosis was demonstrated by cardiac magnetic resonance imaging (CMR) and endomyocardial biopsies (40–42). It is interesting to note that CMR imaging of both T1 and T2 relaxation, reflecting the degree of cardiac fibrosis, was markedly increased in populations of HFmrEF, suggesting that populations of HFmrEF may share this pathophysiological mechanism (42).

Moreover, fibrosis-related biomarkers, including C-terminal propeptide of procollagen type I (PICP) and N-terminal propeptide of procollagen type III (PIIINP), which are regarded as biomarkers of type I and III collagen formation, respectively (43), were significantly higher in the HFmrEF group than in the HFpEF group (44). In a healthy heart, the extracellular matrix (ECM) is mainly composed of collagen with thicker type I collagen fibers (~85–90% of total collagen) and thinner type III collagen fibers (~5–11%) (45). Among them, type I collagen fibers mediate tensile strength, while type III collagen fibers maintain the elasticity of the matrix network (45). In patients with HFmrEF, the PICP and PICP/PIIINP ratios were higher than those in HFpEF patients. This may indicate that there is an equilibrium transfer to type I collagen synthesis in cardiac fibrosis among HFmrEF patients (44), while in HFpEF patients excessive collagen deposition and a shift in collagen type proportion, predominantly a reduction in collagen III, result in increased cardiac stiffness (46).

#### Passive Stiffness

Ventricular stiffness is not only caused by cardiac fibrosis (as described above) but also related to abnormal active relaxation and passive stiffness (F-passive) (37). Titin, a large sarcomeric protein extending from the Z disk to the M-line and encoded by a single gene (47), is the main determinant of F passivity in cardiomyocytes. Different splicing results in different sizes of N2B and N2BA subtypes in the myocardium. Small mammals mainly express N2B titers, while large mammals, including humans, express both stiffer N2B and more flexible N2BA titers (48). Animal models of HFpEF show a shift from N2BA to N2B, which is related to the F-passive increase (49). In contrast, in cardiac biopsies from patients with HFrEF, N2BA subtypes were increased, with no change in total titin levels, indicating a reduction in F-passivity as a result of the transfer from N2B to N2BA subtypes (47).

#### Excitation–Contraction Coupling and Calcium Handling

Excitation–contraction coupling in the heart is a process in which cardiomyocytes are switched from electrical excitation to mechanical force (excitation–contraction). As an indispensable regulator of ECG activity, Ca^2+^ is directly involved in the process of contraction and relaxation of cardiomyocytes. Ca^2+^ released from the sarcoplasmic reticulum (50) acts as a direct activator of myofilaments and will cause cardiac contraction (51). Cardiac relaxation, as a key player in the pathophysiology of HFpEF (52), depends on the reduction of intracellular calcium (Ca^2+^) levels (24), which can be reduced by (1) SERCA, (2) sarcolemmal Na+/Ca^2+^ exchange, (3) sarcolemmal Ca^2+^-ATPase, and (4) mitochondrial Ca^2+^ UniProt (51, 52). Myocardial Ca^2+^ levels are increased in patients with HFpEF, but elevated calcium levels (53) are not associated with an impaired Na+ gradient, in contrast to the case in HFrEF patients who have elevated myocardial [Na+]I (54). The mitochondria also participate in the process of Ca^2+^ concentration regulation because of their capacity to take up cytosolic Ca^2+^ ([Ca^2+^]c) and then use it to regulate energy metabolism, such as in ATP regeneration (55). In rat models of HFpEF cardiomyocytes, free mitochondrial calcium concentrations were higher due to changes in cytosolic and mitochondrial Ca^2+^ processing. In the case of mild mitochondrial dysfunction, the coupling of cytosolic and mitochondrial Ca^2+^ levels may compensate for the myocardial ATP supply (56).

### Exercise Intolerance Associated With Mitochondrial Dysfunction and Cardiac Metabolism

**Figure 2:**
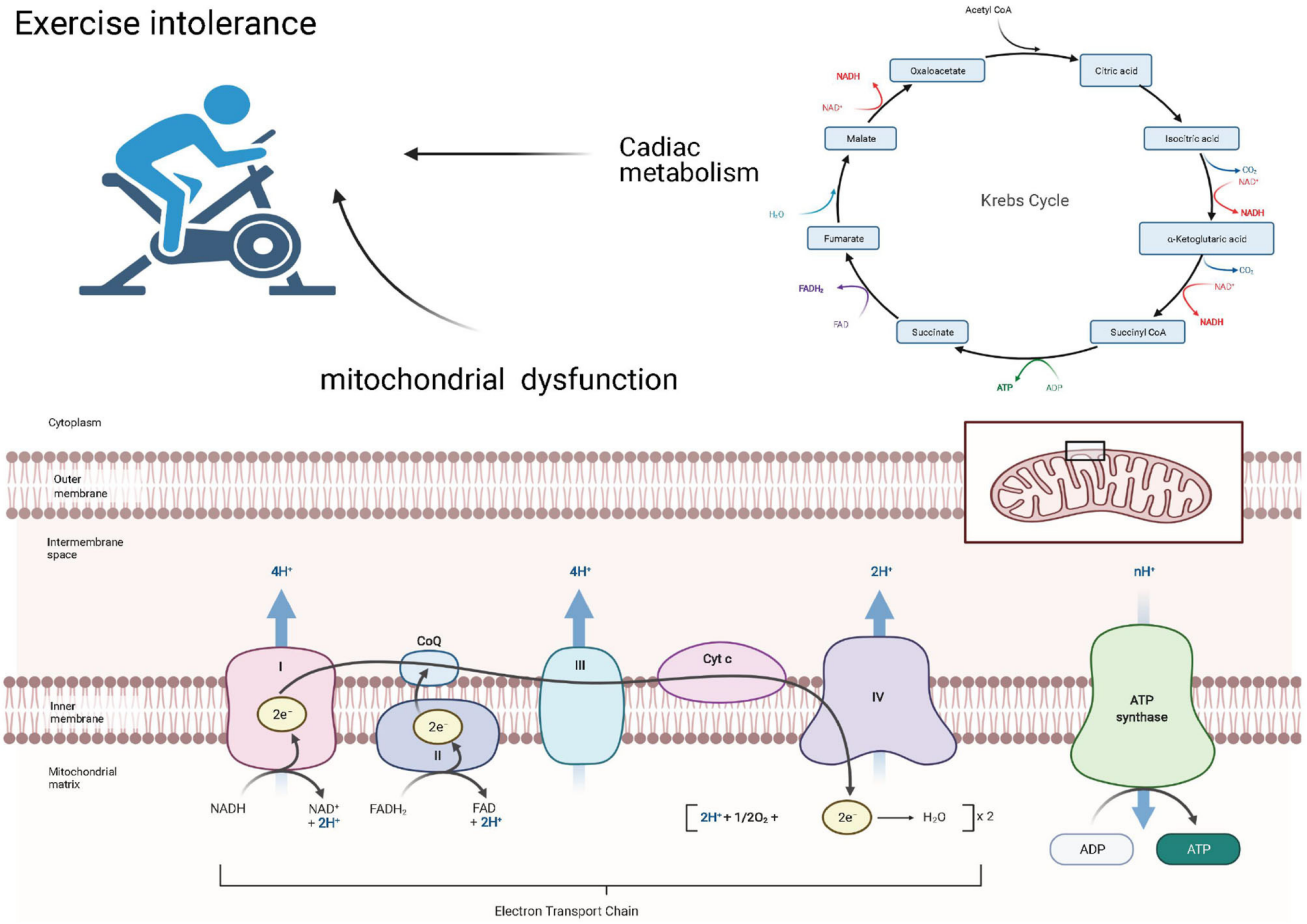
Exercise intolerance associated with mitochondrial dysfunction and cardiac metabolism.

Exercise intolerance is common in the HFpEF population (57) and may be caused by an imbalanced systemic coordination between the cardiac pump, respiratory system, and arterial system (58). Inhaled oxygen will be delivered to the mitochondria of the skeletal muscle and the myocardium, where it can be used to generate ATP to maintain cardiac contraction and relaxation (Figure 2).

#### Mitochondrial Dysfunction

Under normoxic conditions, more than 95% of ATP in the heart is produced by the oxidative phosphorylation of the mitochondria (59), suggesting the vital role this organelle plays in energy production. The structural and functional alterations in this organelle result in an insufficient energy supply in HF patients. Mitochondrial energy production, consisting of the coupling between electron transfer and oxygen uptake, occurs through electron transport chain (ETC) complexes and the phosphorylation of ADP to ATP by F0F1-ATP synthase, also known as complex V (60). A study conducted by Haykowsky et al. demonstrated that, in HFpEF patients, exercise time, peak power output, CO, arterial–venous oxygen difference (A-VO_2_ diff), and peak exercise oxygen consumption (peak VO_2_), a widely validated measure of exercise capacity, were all significantly reduced (61). These findings may suggest a dysfunction between the oxygen delivery and utilization systems. Early theories posited that exercise intolerance and reduced VO_2_ were primarily caused by the absence of a corresponding increase in CO (62, 63). However, recently, more researchers have attributed this discrepancy to peripheral abnormalities (61, 64–66). Among these abnormalities, dysfunction in skeletal muscle mitochondria or cardiomyocyte mitochondria or both appears to be an important pathophysiological contributor (63). Compared with the HFrEF patients, the peak value of A-VO_2_ diff in HFpEF and HFmrEF patients was significantly lower (67), and another trial, conducted by Bhella et al. (64), showed that, in well-compensated HFpEF patients, the cardiac reserve indices were not impaired. A-VO_2_ diff at rest was greater in HFpEF patients than in healthy controls, and exercise training significantly improved the cardiorespiratory fitness in patients with HFpEF. These findings may suggest exercise intolerance in HFmrEF and HFpEF patients predominantly due to peripheral factors, unlike HFrEF, which is caused by impaired cardiac pump performance (67).

The mechanisms of mitochondrial dysfunction are varied (52, 68) and include (i) low activity of the ETC complex (69, 70), (ii) defects in the supermolecular assembly of ETC complexes (71), (iii) increased OS (72), (iv) altered mitochondrial inner membrane (tetralinoleoyl cardiolipin), (v) changes in the tricarboxylic acid cycle (73), (vi) mitochondrial uncoupling (74), (vii) altered energy substrate availability (75), and (viii) abnormal quality control of mitochondrial fission and fusion (76).

#### Cardiac Metabolism

Cardiac metabolism comprises numerous biochemical processes that result in the conversion of substrates for generating energy to meet cell function, growth, and contraction needs. However, due to changes in oxidative substrate utilization and damaged mitochondrial oxidative metabolism, HF patients may exhibit energy deficiency (the ATP levels in HF patients decreased by 30–40% compared with those in healthy hearts) (77).

In Dahl-sensitive rats fed a high-salt diet, changes in energy metabolism during the early stage of HFpEF mainly include an increase in glycolysis, but the increased glycolysis is not accompanied by an increase in glucose oxidation, suggesting that glycolysis and glucose oxidation are decoupled. The fatty acid oxidation rates were in a state of progressive decrease following the high-salt diet (78).

In HFpEF patients, fatty acid oxidation is increased and glucose oxidation is decreased; moreover, there is a state of uncoupling between glucose uptake and glucose oxidation, leading to an increased rate of glycolysis (75). Choi et al. demonstrated that decreased cardiac fatty acid oxidation led to the development of diastolic dysfunction and that targeting acetyl-CoA carboxylase 2, which is a mitochondrial protein directed by its hydrophobic N-terminal leader sequence in the mitochondrial membrane as an essential rate-limiting enzyme in fatty acid metabolism (79), has a positive effect on sustaining mitochondrial fatty acid oxidation, protecting against pathological remodeling, maintaining mitochondrial function, and preventing increases in OS in mice subjected to ATII infusion (80).

Ketone bodies, consisting of β-hydroxybutyrate (β-OHB), acetoacetate, and acetone, are produced predominantly in the liver from FAO-derived acetyl-coenzyme A (CoA) and are transported to extrahepatic tissues for terminal oxidation (81). Alternations in the circulating level of ketone bodies have been shown in HF, which the failing heart relies more on glycolysis and ketone body oxidation (82) as an energy source. This phenomenon has been verified in an HFrEF mouse model, and increased ketone utilization in advanced HF patients was observed (83, 84). Ketone bodies may play a role by inducing adipocytes to uptake fatty acids in the circulation and stimulating cardiomyocytes to uptake glucose, which, in turn, alters the oxidative substrate supply and improves cardiac energy production (85). This is in accordance with a recent study performed by Deng et al. that suggested that increasing myocardial ketone utilization (β-OHB abundance) could significantly mitigate HFpEF phenotypes (86). The mechanism of this improvement was to reduce the acetyl-CoA pool by inhibiting fatty acid uptake and increasing citrate synthase activity to terminate the vicious cycle of mitochondrial dysfunction and inflammation.

## Therapies for HFmrEF and HFpEF

The current ESC guidelines recommend therapies for HFmrEF based on the evidence for HFpEF rather than that for HFrEF. However, in actual clinical practice, the treatment of HFmrEF is closer to that of HFrEF (Table 3).

**Table 3:** Common treatment of patients with HFmrEF and HFpEF.

	**Wang et al. (5)** **In China**		**Fröhlich et al. (4)** **In Northwestern Europe**		**Shiga et al. (87)** **In Japan**		**Farré et al. (88)** **In Spain**	
	**HFmrEF**	**HFpEF**	**HFmrEF**	**HFpEF**	**HFmrEF**	**HFpEF**	**HFmrEF**	**HFpEF**
	***n*** **= 6,825**	***n*** **= 13,497**	***n*** **= 2,086**	***n*** **= 1,146**	***n*** **= 263**	***n*** **= 538**	***n*** **= 504**	***n*** **= 844**
ACEi/ARB (%)	43.0/26.9	28.9/32.0	68.7/18.1	57.5/20.6	27/51	17/45	82.1	70.1
Beta-blockers (%)	75.5	65.7	79.8	72.5	66	48	88.9	71.8
MRAs (%)	72.4	59.2	24.4	20.1	41	35	43.5	26.5
ARNI (%)	2.7	0.7	NA	NA	NA	NA	NA	NA
Loop diuretics (%)	88.6	87.8	60.7	55.4	74	67	83.9	90.5
Digoxin (%)	20.0	15.6	NA	NA	13	9	21.7	22.5
CRT (%)	0.6	0.43	NA	NA	NA	NA	3.17	0.59
ICD (%)	0.9	0.3	NA	NA	NA	NA	5.16	1.31
Statins (%)	73.0	71.1	57.1	58.0	45	26	NA	NA
Anticoagulation therapy (%)	20.9	24.5	46.3	37.9	50	32	47.4	56.4

*ACEI, ACE inhibitor; ARB, angiotensin II receptor blocker; ARNI, angiotensin receptor enkephalinase inhibitor; CRT, cardiac resynchronisation therapy; HFmrEF, heart failure with mid-range left ventricle ejection fraction; HFpEF, heart failure with preserved left ventricle ejection fraction; ICD, implantable cardioverter defibrillator; MRA, mineralocorticoid receptor antagonists; NA, not available*.

### Beta-Blockers

The results of a meta-analysis consisting of 11 clinical trials showed that beta-blockers may halve CV mortality, particularly in the 40–50% LVEF subgroup (*p* = 0.040), regardless of ischemic or non-ischemic etiology. Despite the small number of events, its benefits were similar to those observed in HFrEF, with reductions in both HF-related death and sudden death (89). It is worth noting that the “HFmrEF patients” in the Cleland study accounted for <4% of the entire study population, and the LVEF of most HFmrEF patients was <43% (median value, 40%) (89, 90). Consistent with the above-mentioned findings, the CHART-2 study revealed that, among patients with chronic HF, beta-blockers were related to improved mortality in patients with HFmrEF and HFrEF (*p* = 0.010 and *p* = 0.008, respectively), while there was no significant difference in HFpEF patients (14). Overall, most evidence (14, 89, 91) shows potentially positive effects on short- and long-term outcomes in patients with HFmrEF (92). For HFpEF patients, evidence for the routine use of beta-blockers is inconsistent (93), so the current guidelines do not favor the use of beta-blockers as a general treatment in the HFpEF population (94).

### ACEIs and ARBs

The current guidelines recommend treatment of HFmrEF closer to that of HFpEF compared with HFrEF (95). However, in the 2019 update of the Clinical Practice Expert Consensus Report, it was suggested that candesartan may be considered for HFmrEF patients with symptoms to reduce their risk of heart failure hospitalization and CV death (96).

In the CHARM data, the populations of HFmrEF and HFpEF accounted for 17% (*n* = 1,322) and 26% (*n* = 1,322) of the study patients, respectively (13). Candesartan significantly reduced the primary composite outcome, namely, first HF hospitalization and recurrent HF hospitalization in HFmrEF patients, whereas in HFpEF patients, it did not significantly reduce any outcome. These findings suggest that candesartan improves the outcomes in HFmrEF patients, except that the EF is over 50% (13). Other data from the Swedish HF registry, which identified 42,061 patients, among whom 21% had HFmrEF and 23% had HFpEF, revealed that ACEIs/ARBs are effective in both HFmrEF and HFpEF patients with or without coronary heart disease (97).

In the Hong Kong Diastolic Heart Failure Study (98), the ARB irbesartan and ACEI ramipril did not demonstrate a significant alleviation of the symptoms of HFpEF patients. Another study on irbesartan performed by Massie et al. reached the same conclusion (99). Therefore, neither ACEI nor ARB treatment is recommended in the current guidelines for patients with HFpEF (2).

### Mineralocorticoid Receptor Antagonists

A recent meta-analysis of randomized clinical trials was conducted to explore the efficacy and the safety of spironolactone in the HFmrEF and HFpEF populations. They selected 4,539 patients from 11 randomized controlled trials and concluded that spironolactone treatment may have beneficial effects for HFmrEF and HFrEF patients, namely, reducing hospitalizations and BNP levels, improving NYHA functional classifications (NYHA-FC), and alleviating myocardial fibrosis. Additionally, the only side effects of spironolactone that are of concern are hyperkalemia and gynecomastia (100). The greatest potential benefit from spironolactone treatment has been shown in patients with HFmrEF (45% ≤ LVEF < 50%) compared with other subgroups (LVEF ≥ 50%), especially for those patients with LVEF > 65% and who experienced few positive effects, according to a TOPCAT trial that enrolled patients with HFpEF (LVEF ≥ 45%) (101). They also reported that spironolactone therapy appears to be effective in patients with lower LVEF (LVEF < 50%) in reducing HFH and the primary endpoint (101). In accordance with these conclusions, the Japanese Cardiac Registry of Heart Failure in Cardiology reported that, at a mean follow-up of 2.2 years, spironolactone significantly reduced the compound mortality of all-cause death or heart failure rehospitalization (102). Another retrospective study from China came to the same conclusion, namely, spironolactone markedly reduced rehospitalization and the incidence of the primary composite outcome of all-cause death in HFmrEF patients (103). Meanwhile, research that limited the TOPCAT analysis to patients in the Americas, consisting of 1,644 HFpEF patients, showed that, compared with placebo, spironolactone treatment markedly reduced the clinical symptoms of congestion, and lower congestion was independently associated with a higher quality of life and better outcomes (104).

### Sacubitril/Valsartan

Sacubitril/valsartan has shown better effects in reducing the risks of death and hospitalization in patients with HFrEF than enalapril (105), but whether it has similarly beneficial effects in HFmrEF and HFpEF populations is still unclear. Therefore, following the PARADIGM-HF trial in HFrEF patients (105), the 2019 PARAGON trial screened 4,796 patients with LVEF ≥ 45% and symptomatic HF, accompanied by elevated levels of natriuretic peptides and cardiac structural abnormalities, and then assessed the clinical outcomes of patients treated with sacubitril–valsartan or valsartan (106). During a median follow-up of 35 months, Solomon et al. found that there was no significant benefit of sacubitril/valsartan on the primary composite outcome of the total hospitalization for HF and death from CV causes. However, among one of the pre-specified subgroup analyses, those with LVEF in the lower part (45–57%) of the range were more likely to benefit from sacubitril/valsartan. The rates observed in the subgroup below the median were similar to those observed in the PARADIGM-HF trial, which was focused on the HFrEF population (106). Meanwhile, patients treated with sacubitril/valsartan were more likely to suffer hypotension but less likely to have elevated creatinine and potassium concentrations compared with those in the valsartan-alone group. A recent meta-analysis of a total of 5,503 patients from six studies reported that sacubitril–valsartan may play an effective and safe role in improving the clinical symptoms and reducing HFH in HFmrEF and HFpEF patients (107).

### Statins

Statins have been shown to reduce the morbidity and the mortality in CVD and other related diseases, such as MI, stroke, and revascularization (108–110), but their effectiveness in HFmrEF and HFpEF is still inconclusive. An observational study from the Swedish Heart Failure Registry performed by Alehagen et al. proposed a possible benefit of statins in reducing all-cause mortality and CV hospitalization in patients with HFmrEF and HFpEF. Meanwhile, statins had a positive effect in controlling mortality and the combined endpoint of all-cause mortality (111).

### Sodium–Glucose Cotransport Protein 2 Inhibitor

The 2019 Clinical Practice Expert Consensus Report proposed that sodium–glucose cotransport protein 2 (SGLT2) inhibitors (canagliflozin and dapagliflozin) may be considered for patients with T2DM and those with CV disease or high CV risks to prevent or delay the onset of hospitalizations associated with HF (96). SGLT2 inhibitors can maintain electrophysiological stability (cardiomyocyte Na+/H exchanger inhibition) (112) and cardiac hemodynamics (113), inhibit cardiac fibrosis (114), and improve myocardial systolic and diastolic function (115). Thus, in theory, more HFpEF patients could benefit from SGLT2 inhibitors. In the EMPA-REG OUTCOME® trial, patients with T2DM were at a high risk of CV events, and empagliflozin was associated with a 38% decrease in CV-related death, a 35% reduction in HF hospitalization, and a 32% reduction in all-cause mortality (116). In a *post-hoc* analysis of DECLARE-TIMI 58, among HFrEF populations who had a pre-specified EF cutoff point of <45%, including the population that is now defined as HFmrEF with an ejection fraction of 40–49%, dapagliflozin showed a greater benefit in HF hospitalization and CV death than patients with an EF known to be ≥45% or those without EF history. Furthermore, a similar reduction in HF hospitalization was found in patients with HF regardless of EF, but a greater reduction with dapagliflozin in CV death was only found in HFrEF patients (117). Although some animal experiments have demonstrated the efficacy of SGLT2 inhibitors in HFpEF models, including improving cardiac diastolic function (118), cardiac hypertrophy, and tissue fibrosis (119, 120), there is still insufficient evidence to extend these studies to clinical practice in HFpEF or even HFmrEF patients.

### Vericiguat

In HFpEF, the intracellular NO-cGMP-PKG signal cascade is disturbed (121), and downregulating NO-cGMP-PKG signaling may be attributed to cardiomyocyte hypertrophy and stiffness in HFpEF. Vericiguat, as a soluble guanylate cyclase (SGC) stimulator, enables the direct generation of cGMP and maintains the sensitivity of SGC to endogenous NO (122), which may have a positive effect on the HFpEF population. In the phase 2 SOCRATES-PRESERVED study, vericiguat, at a study dose of 1.25/2.5/5/10 mg for 12 weeks in HFpEF (LVEF ≥ 45%) patients, did not reduce the primary endpoint of NT-proBNP and left atrial volume levels compared with the placebo; however, the quality of life of HFpEF patients, as assessed by the Kansas City Cardiomyopathy Questionnaire (KCCQ), was improved after receiving the two higher doses of vericiguat, and the tolerability of vericiguat was also confirmed (123). Another study published in 2020 showed that, in patients with HFpEF (LVEF ≥ 45%), vericiguat (15 and 10 mg/day) did not significantly improve the KCCQ Physiological Limits Score or the 6-min walking distance (6MWD) after 24 weeks of treatment (124). The subjects (HFpEF patients) in both of the studies described above included a portion of the population that is now defined as HFmrEF (EFs 40–49%). The hypothesis cannot be confirmed for this recent trial, possibly due to the following: (a) compared with the former trail, the subjects in a recent study had higher baseline KCCQ scores, a higher percentage of NYHAC II patients, and a lower proportion of NYHAC III patients, (b) the placebo group showed a higher improvement score than the former group, and (c) NO may not be a key regulatory factor in HFpEF progression. The phase 3 VICTORIA trial (125) investigated the efficacy of vericiguat in patients with HFrEF (LVEF ≤ 45%). Studies have shown that vericiguat reduces the incidence of death from CV causes or hospitalization for HF, but it does not appear to be effective in patients with LVEF of 40–45% (125, 126). This suggests that vericiguat may be more effective in patients at a high risk of HF decompensation.

### Cardiac Contractility Modulation

Cardiac systolic modulation (CCM) is administered through an implantable pulse generator. It can enhance the contractility of the right ventricle by transmitting the CCM pulse to the right ventricle during the absolute refractory period (127). The 2019 expert consensus suggested that CCM may be considered for patients with HFrEF (LVEF 25–45%) and a narrow QRS complex (<130 ms) (96).

CCM has been shown to improve the quality of life, LVEF, and NYHAC (115) during a 24-month follow-up performed by Muller et al. (128). This is consistent with a study performed by Yu et al., who indicated that CCM improved both global and regional LV contractility, which may contribute to the reversal of LV remodeling and improved systolic function as well as improved performance in the 6MWD and NYHA-FC (129). These findings have recently been supported by another randomized FiX-HF 5C trial (130), including a total of 160 patients with NYHA-FC symptoms, QRS duration <130 ms, and LVEF ≥ 25 and ≤ 45%. It showed increases in peak oxygen uptake as well as in Minnesota Living With Heart Failure Questionnaire (*P* < 0.001), NYHAC (*P* < 0.001), and 6MWD (*P* = 0.02) scores. This study further confirmed that, in the affected population, cases with an EF between 35 and 45%, including some cases of HFmrEF (EFs 40–49%), gain greater clinical benefits than those with EF <35% (130). These findings show that CCM may be considered for HF patients with higher EFs, especially for those with pathogenesis that includes Ca^2+^ processing, cytoskeletal stability, changes in ECM, and excessive activation of the autonomic nervous system (131), all of which are related to the pathophysiological mechanism of HFpEF (132).

## Conclusions

The emergence of the concept of HFmrEF has achieved its aim to draw increasing attention from researchers on its mechanism, treatments, and clinical characteristics. However, considering current evidence and pathophysiology, the effective therapeutics for both HFmrEF and HFpEF patients are still insufficient, especially in the former. Compared with the HFpEF patients, the HFmrEF patients are more similar to HFrEF patients with respect to clinical characteristics, such as age, sex, systolic blood pressure, and ischemic etiology as well as clinical biomarkers such as hs-TnT and hs-CRP. The pharmacological treatment in HFmrEF has also followed almost the same strategies as those in HFrEF. However, with regard to the clinical prognosis and some aspects of the pathophysiology mechanism, HFmrEF and HFpEF share some commonalities. A recent study also demonstrated that patients whose LVEF deteriorated or improved to or remained stable at mid-range levels show a different prognosis, and patients with HFmrEF can even convert into HFpEF or HFrEF. Therefore, whether HFmrEF is an intermediate status, what the interaction is between HFmrEF and HFpEF or HFrEF, what the exact mechanism of HFmrEF is, and how this mechanism affects prognosis still warrant further study and exploration to improve the outcome in HFmrEF patients.

## Author Contributions

HZ, YN, YT, DX, and QZ contributed to the review design. PL, HZ, and JZ searched and collected the relative literature. PL, HZ, YN, and QZ draw the manuscript. PL, HZ, JZ, YN, YT, DX, and QZ revised and finalized the manuscript. All authors contributed to manuscript revision, read, and approved the submitted version.

## Funding

This project was partly supported by the Science and Technology Program of Guangzhou (201804010086 and 201707020012; QZ and DX), the National Natural Science Foundation of China (82070403 and 81770386; QZ), the Frontier Research Program of Guangzhou Regenerative Medicine and Health Guangdong Laboratory (2018GZR110105001; QZ), and the Youth Science and Technology Innovation Talent of Guangdong TeZhi Plan (2019TQ05Y136; QZ).

## Conflict of Interest

The authors declare that the research was conducted in the absence of any commercial or financial relationships that could be construed as a potential conflict of interest.

## Publisher's Note

All claims expressed in this article are solely those of the authors and do not necessarily represent those of their affiliated organizations, or those of the publisher, the editors and the reviewers. Any product that may be evaluated in this article, or claim that may be made by its manufacturer, is not guaranteed or endorsed by the publisher.
